# Association between body mass index and survival in Taiwanese heart failure patients with and without diabetes mellitus

**DOI:** 10.1097/MD.0000000000028114

**Published:** 2021-12-03

**Authors:** Yu Ying Lu, Victor Chien Chia Wu, Pao-Hsien Chu, Chien-Te Ho, Chieh-Yu Chang

**Affiliations:** Division of Cardiology, Chang Gung Memorial Hospital, Linkou, Taoyuan, Taiwan.

**Keywords:** body mass index, diabetes mellitus, heart failure, obesity paradox

## Abstract

Body mass index (BMI) is positively associated with survival in heart failure (HF) patients with reduced ejection fraction (HFrEF). However, emerging evidence shows that this benefit may not exist in diabetic patients with HFrEF. As this relationship has not been investigated in Asian patients, the aim of this study was to examine the association between obesity and outcomes in HrEFF patients with and without diabetes mellitus (DM), and discuss the potential underlying mechanisms.

The analysis included 900 patients with acute decompensated HF from the Taiwan Society of Cardiology-Heart Failure with Reduced Ejection Fraction Registry, of whom 408 had DM (45%). The association between BMI and all-cause mortality was examined using multivariate Cox proportional hazards regression after adjusting for covariates and Kaplan–Meier survival analysis. Echocardiography parameters were also analyzed in patients with different BMI and DM status.

After adjusting for confounding factors, BMI was a significant independent predictive factor for all-cause mortality in the non-diabetic patients (hazard ratio [HR], 0.88; 95% confidence interval [CI], 0.81–0.95) and in Kaplan–Meier survival analysis (log-rank test, *P* = .034). For diabetic patients, BMI was not a significant predictive factor for all-cause mortality (HR, 0.96; 95% CI, 0.90–1.02) and in Kaplan–Meier survival analysis (log-rank test *P* = .169). Both DM (47.8 vs 45.4 mm, *P* = .014) and higher BMI (48.6 vs 44.9 mm, *P* < .001) are independently associated with higher left atrial size. Patients with a higher BMI had a lower proportion of severe mitral regurgitation (10.0% vs 14.1%, *P* < .001).

In non-diabetic patients with HFrEF, BMI was a significant predictor of survival. However, in diabetic patients with HF, BMI was not a significant predictor of survival. Diastolic dysfunction in patients with DM and obesity may have played a role in this finding.

## 1 Introduction

Body mass index (BMI) has been shown to be an independent risk factor for cardiovascular morbidity and developing heart failure (HF). However, for patients with heart failure with reduced ejection fraction (HFrEF), a large body of evidence supports the “obesity paradox” phenomenon, in which BMI is positively associated with survival.^[1]^ This may cause overweight or obese patients to avoid losing weight and possibly lead to detrimental health effects, especially for those comorbid with type 2 diabetes mellitus (DM). There is strong and consistent evidence that managing body weight can delay the progression from prediabetes to type 2 DM, and that it may be beneficial in the treatment of type 2 DM.^[2]^ The possible benefits of weight reduction in such patients are important. Furthermore, emerging evidence suggests that high BMI confers no paradoxical survival benefit in patients with both HFrEF and DM,^[3,4]^ although this has yet to be investigated in Asian populations. In recent decades, there has been a dramatic increase in the number of people with DM in Asia, and more than 60% of diabetic patients now live in this region.^[5]^ The aim of this study was to evaluate the association between BMI and survival in Taiwanese HFrEF patients with and without DM.

## 2 Materials and methods

### 2.1 Study data and patients

**Figure 1:**
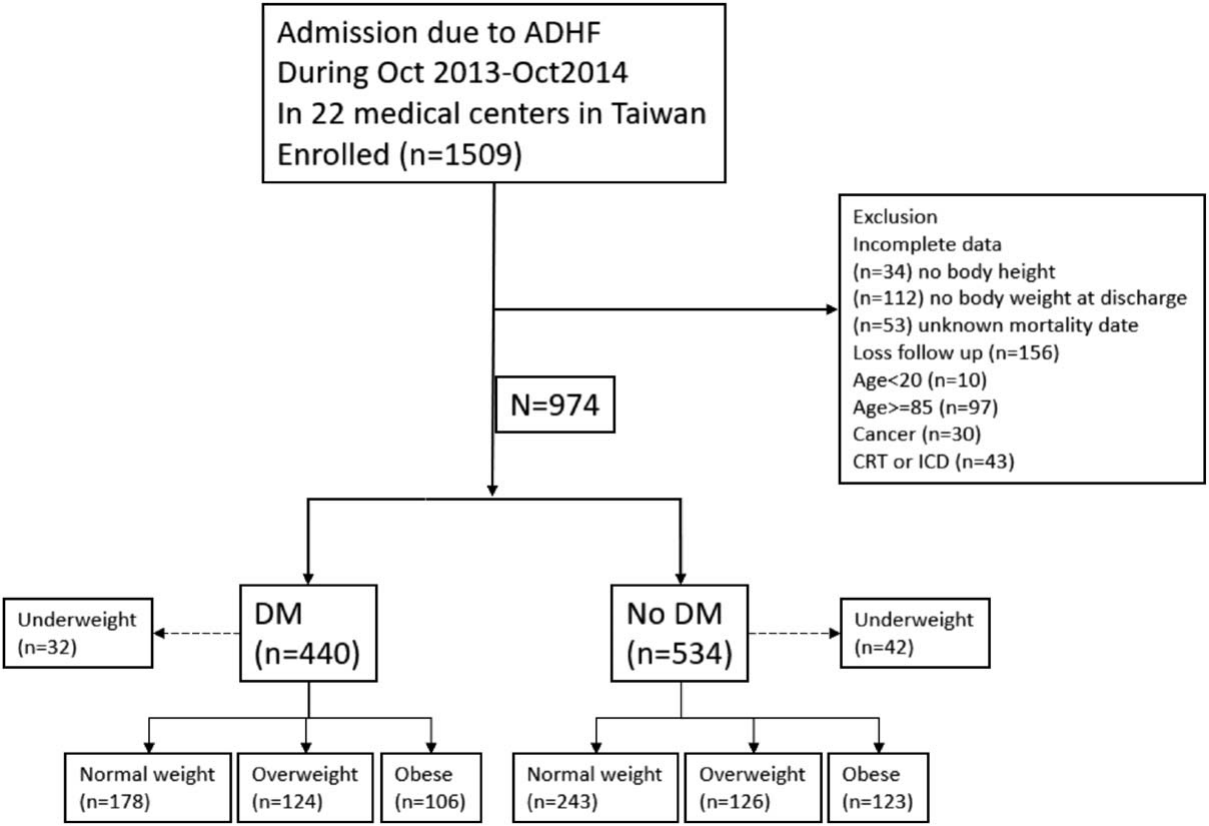
Study design. ADHF = acute decompensated heart failure, CRT = cardiac resynchronized therapy, DM = diabetes mellitus, ICD = implantable cardioverter-defibrillator.

We conducted a secondary analysis of the Taiwan Society of Cardiology-Heart Failure with Reduced Ejection Fraction registry. The rationale, design, and definition of the diagnostic criteria have been described in detail previously.^[6]^ In brief, it was a multi-center study that prospectively investigated the prognosis of patients with HFrEF in Taiwan. Patients were eligible for enrollment if they had been hospitalized for acute worsening of HF between October 2013 and October 2014 at pre-specified 22 medical centers throughout Taiwan. These patients were followed up after discharge for a median of 1 year on the outcome of mortality. We further divided the participants into 3 groups by BMI at discharge: 18.5 to 24.0 kg/m^2^ (normal weight), 24.1 to 27.4 kg/m^2^ (overweight), and ≥27.5 kg/m^2^ (obesity), according to the evident-based guidelines on adult obesity and management, published by Taiwan Health Promotion Administration.^[7]^ Patients were excluded if age <20 years old or age ≥85 years old, underweight (BMI < 18.5), had malignancy, received cardiac resynchronized therapy or implantable cardiac defibrillators. Chronic kidney disease (CKD) in this study was defined by past medical records or estimated glomerular filtration rate <60 mL/min/1.73 m^2^ for more than 3 months during follow-up. Smoking was defined by both current and former smokers. Echocardiography parameters including left atrial (LA) diameter, left ventricular end-diastolic diameter (LVEDD), left ventricular (LV) mass, E/A ratio, and severity of mitral regurgitation were collected. Electrocardiography parameters, including heart rate, QRS duration, and QTc duration, were also collected. The outcome of interest was all-cause mortality. The patients were followed up at outpatient departments every 6 months to evaluate their clinical conditions and laboratory tests. Those with missing clinical data or those who failed to complete 1 year of follow-up were also excluded from the analysis. The date of mortality was verified based on the medical records or phone contact. The study design flowchart and patient enrollment are shown in Figure 1. This study complied with the Declaration of Helsinki, and the Joint Ethics Committee approved the study protocol. Informed consent was obtained from all the study subjects. The included patients were further divided into 2 groups for analysis: those with DM and those without DM. DM was defined according to the World Health Organization diagnostic criteria^[8]^ or the use of hypoglycemic medications. Baseline diabetes data were available for all the participants.

### 2.2 Statistical analysis

Basic characteristics and demographic characteristics of the patients in the DM and non-DM groups were compared using either analysis of variance or the chi-square test and reported as mean ± standard deviation or percentage for continuous and categorical variables, respectively. Comparisons of continuous variables among BMI groups were performed using analysis of variance followed by the Fisher least significant difference test or the Kruskal–Wallis test for post-hoc pairwise comparison as appropriate. A χ^2^ test was used to compare categorical variables among BMI groups. Kaplan–Meier analysis was used to assess the association between the 3 BMI categories and mortality. Survival curves were compared using the log-rank test. Cox proportional hazard regression analysis was used to assess univariate and multivariate associations of BMI (as a continuous variable) with mortality, adjusting for potential confounders including age, New York Heart Association (NYHA) functional class, atrial fibrillation, CKD, and beta-blocker use at discharge. Echocardiography and electrocardiography parameters, including LA diameter, LVEDD, ejection fraction, LV mass, heart rate, QRS duration, and QTc duration in patients with and without DM, with BMI ≤24 and BMI >24 were also compared using 2 sample *T* test, and severity of mitral regurgitation was compared using the chi-square test. All statistical analyses were performed with R software version 3.5.2 (The R Foundation for Statistical Computing Platform, Vienna, Austria), and a *P* value <.05, was considered statistically significant.

## 3 Results

**Figure 2:**
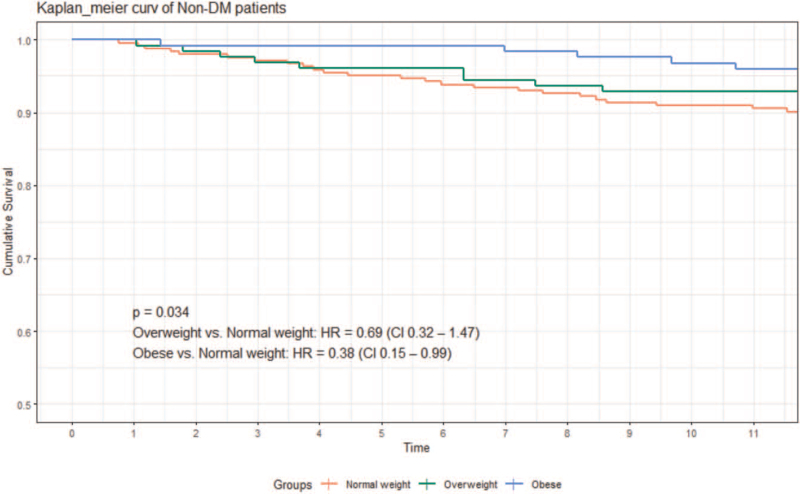
Kaplan–Meier event-free curves for all-cause mortality in the non-diabetic patients based on discharge BMI groups. BMI = body mass index, DM = diabetes mellitus.

**Figure 3:**
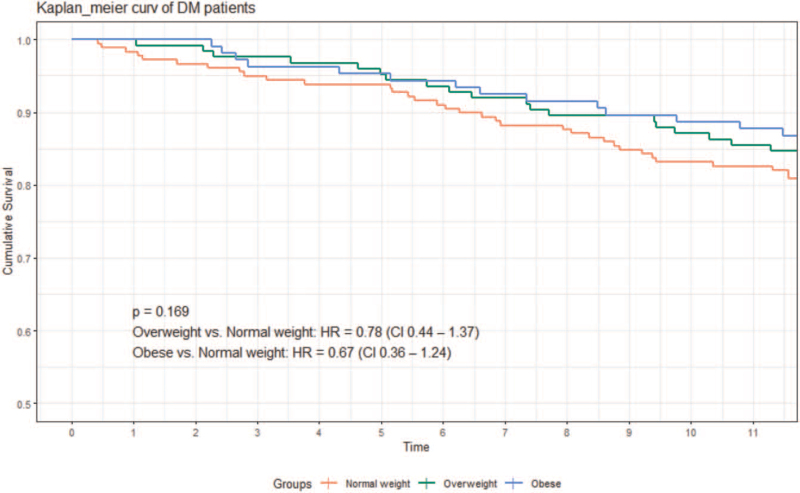
Kaplan–Meier event-free curves for all-cause mortality in the diabetic patients based on discharge BMI groups. BMI = body mass index, DM = diabetes mellitus.

Overall, 1509 patients were enrolled in this study, of whom 199 were excluded because of incomplete data collection (34 without body height, 112 without body weight at discharge, and 53 without mortality date), 156 were lost to follow-up within 1 year, 97 were aged ≥85 years, 10 were aged <20 years, 74 had BMI < 18.5, 30 had a history of cancer, and 43 had implantable defibrillators or re-synchronized therapy. The remaining 900 patients were included in the analysis, of whom 408 had DM. The baseline characteristics of the DM and non-DM groups are shown in Table 1. Patients with DM had more comorbid conditions than those without DM, including older age, ischemic heart disease, CKD, higher baseline creatinine level, higher glycohemoglobin level, and less use of angiotensin-converting enzyme inhibitor or angiotensin receptor blocker at discharge. The non-DM group had a lower average age, lower mean ejection fraction, and more male patients. There was no significant difference in discharge BMI distribution between the 2 groups. Table 2 shows comparisons of sample characteristics among normal-weight, overweight, and obese patients according to the presence or absence of diabetes. Among patients without DM, the obese patients were younger, had higher rates of beta-blocker prescriptions at discharge, and had a lower prevalence of atrial fibrillation. Among the patients with DM, obese patients were also younger and had a higher rate of beta-blocker prescription, but normal-weight patients had less atrial fibrillation. A total of 108 deaths occurred after 365 days of follow-up, including 69 (63.9%) in the DM group and 39 (36.1%) in the non-DM group. Kaplan–Meier analysis showed that the risk of death differed among the 3 BMI groups without DM (log-rank test, *P* = .034), but not among the 3 BMI groups with DM (log-rank test *P* = .169) (Figs. 2 and 3). Table 3 shows the results of Cox proportional hazard regression analysis of univariate and multivariate associations between BMI (as a continuous or categorical variable, respectively) with mortality, after adjusting for confounders including age, sex, NYHA functional class III and IV at discharge, CKD, atrial fibrillation, history of stroke, heart failure etiology, ejection fraction, and beta-blocker use at discharge. BMI was a significant independent predictive factor for all-cause mortality in non-diabetic patients (hazard ratio [HR], 0.88; 95% confidence interval [CI], 0.80–0.97). Other predictive factors for mortality included NYHA functional class III or IV at discharge and a lower ejection fraction. BMI was not a significant predictive factor for all-cause mortality in patients with diabetes (HR, 0.96; 95% CI, 0.91–1.02). Among the covariates included in this analysis, older age, male sex, NYHA functional class III or IV at discharge, and CKD were associated with an increased risk of all-cause mortality in patients with DM. Table 4 shows the difference in echocardiographic and electrocardiographic parameters between patients with and without DM and BMI ≤ 24 kg/m^2^ and BMI > 24 kg/m^2^. DM patients had significantly larger LA diameter (47.8 vs 45.4 mm, *P* = .014) and LVEDD (63.9 vs 60.6 mm, *P* = .011). The proportion of severe mitral regurgitation was also significantly higher in patients with DM (14.9% vs 8.9%, *P* = .013). Patients with BMI > 24 kg/m^2^ had higher LA diameter (48.6 vs 44.9 mm, *P* < .001) and LV mass (323.9 vs 269.5 g, *P* < .001). However, patients with a BMI > 24 kg/m^2^ had less severe mitral regurgitation (10.0% vs 14.1%, *P* < .001).

**Table 1:** Baseline characteristics of the patients with systolic heart failure according to the presence of diabetes.

	Non-DM	DM	*P* value
No. of patients	492	408	
Age (mean (SD)), year	59 (16.0)	63 (12.5)	<.001
Male, No. (%) of patients	392 (79.7)	289 (70.8)	.003
BMI (mean (SD)), kg/m^2^	24.8 (4.4)	25.2 (4.4)	.104
Glycohemoglobin (mean (SD)) (%)	5.9 (0.3)	7.8 (1.2)	<.001
Group, No. (%) of patients			.173
Normal weight	243 (49.4)	178 (43.6)	
Overweight	126 (25.6)	124 (30.4)	
Obese	123 (25.0)	106 (26.0)	
CKD, No. (%) of patients	95 (19.3)	158 (38.7)	<.001
Atrial fibrillation, No. (%) of patients	133 (27.0)	91 (22.3)	.120
NYHA Fc, No. (%) of patients			.950
I/II	371 (75.4)	306 (75.0)	
III/IV	121 (24.6)	102 (25.0)	
Ischemic heart, No. (%) of patients	166 (33.7)	229 (56.1)	<.001
Smoke, No. (%) of patients	271 (55.1)	215 (52.7)	.517
Stroke, No. (%) of patients	36 (7.3)	45 (11.0)	.069
COPD, No. (%) of patients	43 (8.7)	31 (7.6)	.618
Ejection fraction (mean (SD)) (%)	27.23 (8.14)	28.55 (7.96)	.014
Creatinine (mean (SD)), mg/dL	1.55 (1.81)	2.11 (2.03)	<.001
Discharge medications, No. (%) of patients			
Beta-blocker (%)	309 (62.8)	258 (63.2)	.124
ACEi/ARB (%)	347 (70.5)	231 (56.6)	<.001

**Table 2:** Comparisons of sample characteristics among normal-weight, overweight, and obese patients according to the presence or absence of diabetes.

	DM Group				Non-DM Group			
	Normal	Overweight	Obese	*P*	Normal	Overweight	Obese	*P*
No. of patients	178	124	106		243	126	123	
Age (mean (SD)), year	65.7 (10.8)	64.2 (11.3)	55.5 (13.7)	<.001	63.0 (15.5)	57.9 (14.4)	50.8 (15.6)	<.001
Male, No. (%) of patients	115 (64.6)	90 (72.6)	84 (79.2)	.028	186 (76.5)	103 (81.7)	103 (83.7)	.216
BMI (mean (SD)), kg/m^2^	21.6 (1.5)	25.4 (0.9)	31.0 (3.6)	<.001	21.4 (1.6)	25.5 (1.0)	30.6 (4.0)	<.001
CKD, No. (%) of patients	77 (43.3)	48 (38.7)	33 (31.1)	.128	51 (21.0)	22 (17.5)	22 (17.9)	.645
Atrial fibrillation, No. (%) of patients	27 (15.2)	38 (30.6)	26 (24.5)	.005	81 (33.3)	26 (20.6)	26 (21.1)	.008
NYHA Fc, No. (%) of patients				.753				.155
I/II	135 (75.8)	90 (72.6)	81 (76.4)		177 (72.8)	103 (71.7)	91 (74.0)	
III/IV	43 (24.2)	34 (27.4)	25 (23.6)		66 (27.2)	23 (18.3)	32 (26.0)	
IHD, No. (%) of patients	107 (60.1)	71 (57.3)	51 (48.1)	.137	87 (35.8)	46 (36.5)	33 (26.8)	.172
Smoke, No. (%) of patients	86 (48.3)	66 (53.2)	63 (59.4)	.191	128 (52.7)	70 (55.6)	73 (59.3)	.476
Stroke, No. (%) of patients	16 (9.0)	16 (12.9)	13 (12.3)	.506	23 (9.5)	9 (7.1)	4 (3.3)	.097
COPD, No. (%) of patients	13 (7.3)	13 (10.5)	5 (4.7)	.253	28 (11.5)	9 (7.1)	6 (4.9)	.08
Ejection fraction (mean (SD)) (%)	29.0 (7.4)	27.9 (8.2)	28.5 (8.5)	.475	27.2 (8.1)	27.7 (8.1)	26.7 (8.3)	.621
Creatinine (mean (SD)), mg/dL	2.2 (2.1)	2.1 (1.9)	2.0 (2.1)	.869	1.4 (1.2)	1.7 (2.1)	1.6 (2.3)	.278
Discharge medications, No. (%) of patients								
Beta-blocker	103 (57.9)	76 (61.3)	79 (74.5)	.016	143 (58.8)	75 (59.5)	91 (74.0)	.054
ACEi/ARB	94 (52.8)	68 (54.8)	69 (65.1)	.116	165 (67.9)	90 (71.4)	92 (74.8)	.626

**Table 3:** Cox proportional hazard regression analyses of Univariate and multivariate associations of body mass index (as a continuous variable) with all-cause mortality in the DM and non-DM groups.

	DM Group			Non-DM Group		
	Hazard ratio	*P* value	95% CI	Hazard ratio	*P* value	95% CI
Univariate model						
Body mass index	0.96	.214	0.91–1.02	0.88	.008	0.80–0.97
Multivariate model						
Body mass index	0.96	.168	0.90–1.02	0.88	.002	0.81–0.95
Female	0.53	.023	0.30–0.92	1.83	.07	0.95–3.52
Age	1.03	.003	1.01–1.06	1.01	.391	0.99–1.03
Ischemic etiology	1.13	.612	0.70–1.81	0.77	.441	0.40–1.49
NYHA Fc, III/IV	1.98	<.001	1.33–2.94	2.20	.01	1.21–3.99
Chronic kidney disease	1.66	.010	1.13–2.46	1.36	.412	0.65–2.84
Atrial fibrillation	0.91	.675	0.59–1.40	0.74	.409	0.36–1.51
Beta-blocker use at discharge	0.76	.175	0.52–1.13	0.70	.245	0.39–1.28
Ejection fraction	0.98	.17	0.95–1.01	0.94	.002	0.91–0.98

**Table 4:** Differences of echocardiography and electrocardiography parameters between heart failure patients (right: with and without diabetes mellitus) (left: with BMI ≤ 24 and BMI > 24).

	Non-DM *N* = 492	DM N = 408	*P* value	BMI ≤24 kg/m^2^ N = 421	BMI > 24 kg/m^2^ N = 479	*P* value
LA diameter (mean (SD), m)	45.36 (9.32)	47.83 (18.88)	.014	44.94 (9.00)	48.57 (19.78)	<.001
E/A ratio (mean (SD))	1.65 (1.04)	1.56 (0.90)	.507	1.68 (0.95)	1.53 (1.01)	.266
LV mass (mean (SD), g)	290.3 (109.8)	300.7 (108.2)	.386	269.5 (98.5)	323.9 (112.8)	<.001
LVEDD (mean (SD), m)	60.61 (19.55)	63.91 (20.39)	.011	61.66 (21.77)	63.22 (18.12)	.227
Ejection fraction (mean (SD)), %	27.23 (8.14)	28.55 (7.96)	.014	28.14 (8.13)	27.63 (8.30)	.333
Mitral regurgitation (n, (%))			.013			<.001
Mild	266 (54.1)	192 (47.1)		185 (43.9)	273 (57.0)	
Moderate	182 (37.0)	155 (38.0)		177 (42.0)	158 (33.0)	
Severe	44 (8.9)	61 (14.9)		59 (14.1)	48 (10.0)	
Heart rate (mean (SD), bpm)	97.74 (23.00)	98.91 (26.91)	.477	97.37 (24.34)	99.47 (26.09)	.201
QRS duration (mean (SD)), ms	112.03 (33.74)	110.57 (29.61)	.479	111.02 (32.05)	111.45 (30.98)	.837
QTc duration (mean (SD)), ms	470.15 (55.75)	464.69 (54.30)	.129	470.47 (52.55)	463.63 (57.33)	.056

## 4 Discussion

**Figure 4:**
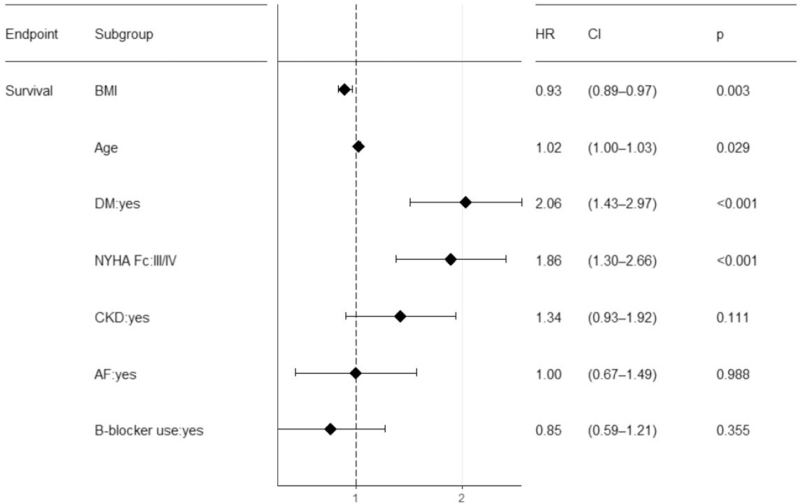
Hazard ratios of different variables associated with mortality. AF = atrial fibrillation, BMI = body mass index, CKD = chronic kidney disease, DM = diabetes mellitus, NYHA = New York Heart Association Functional Class.

In this study, for patients with HFrEF in Taiwan, there was a reverse association between BMI and all-cause mortality in the non-DM group (Fig. 2). However, in the DM group, this association was not significant (Fig. 3). The protective effects of BMI in patients with HFrEF have been the focus of intense research. Various mechanisms have been proposed to explain this paradox, including possible greater metabolic reserve, a more attenuated response to the renin-angiotensin-aldosterone system, and more tolerable cardioprotective medications.^[9,10]^ Our results are in line with emerging evidence that showed this reverse association may not exist in patients with HFrEF with coexisting DM.^[1,3,4,11,12]^ If we examine it closely, what has changed is the obesity part in the DM group seems to lose their survival protective effect. Such a result has also been observed in previous studies.^[3,11]^ The underlying mechanism is still unknown. Adamopoulos et al suggested that the presence of DM may be a much stronger predictor of outcomes than obesity *per se*.^[4]^ Our analysis also confirmed that HF patients with diabetes had a higher mortality rate at 1 year (18% vs 10%, *P* < .05) and DM was a stronger predictor for death than BMI (Fig. 4, DM, hazard ratio: 2.06, CI: 1.43–2.97; BMI, hazard ratio: 0.93, CI: 0.89–0.97). To further investigate the underlying etiology, we performed a cross-analysis of echocardiographic and electrocardiographic parameters in patients with and without DM and in patients with BMI ≤ 24 kg/m^2^ and BMI > 24 kg/m^2^. Our analysis showed that HF patients with DM had higher mean LA size, LVEDD, and more severe mitral regurgitation (Table 4, left). Patients with a higher BMI had higher LA size, LV mass, and less mitral regurgitation (Table 4, right). The higher proportion of severe mitral regurgitation in patients with DM might be attributed to its higher ischemic etiology. Interestingly, patients with a higher BMI protected them from severe mitral regurgitation. Not all the information on the etiologies of severe mitral regurgitation were available, and this is a limitation of our study. Mitral valve prolapse has been reported to be inversely associated with BMI.^[13]^ The reverse association between BMI and severe mitral regurgitation in patients with HF might explain the reason for better survival in obese patients. However, both DM and higher BMI are independently associated with higher atrial size, which is correlated with higher atrial pressure and diastolic dysfunction, regardless of mitral regurgitation severity. “Diabetic cardiomyopathy” may also play a part in this result; both obesity and glucose metabolism disorders are independently associated with left ventricular concentric remodeling and have a negative impact on diastolic function independently, which may worsen when these factors coexist together and further affects the outcome.^[14–17]^ To the best of our knowledge, this is the first study to report this phenomenon in an Asian population. Obese HF patients with less severe mitral regurgitation are also an interesting finding in our analysis and may partly explain the obesity paradox in HF patients.

In Asia, the prevalence of patients with concomitant DM and HFrEF is growing exponentially.^[18,19]^ Owing to the increasing burden of obesity, new weight management strategies for these patients are urgently needed. Currently, there is no clear consensus regarding the recommendation of weight management in patients with established HFrEF.^[20]^ The American College of Cardiology and American Heart Association HF clinical practice guidelines for adults^[21]^ do not specifically comment on the management of HFrEF in obese patients, whereas the European Society of Cardiology^[22]^ recommends weight reduction for more advanced obesity (BMI 35–45 kg/m^2^) to manage the symptoms and exercise capacity. For Asian patients, the BMI cutoff values are lower than in other populations (BMI ≥ 27.5 kg/m^2^); therefore, these guidelines may not be applicable to Asian populations, and ideal weight management strategies for these patients remain uncertain.

While obesity and DM are well-established risk factors for HF, the potential benefit of weight loss to either prevent or treat the condition in obese patients remains incompletely studied.^[23]^ Increasing evidence has shown the beneficial effects of weight loss in DM patients,^[24,25]^ and some studies have shown the possible positive effect of bariatric surgery in HF patients.^[26–29]^ Weight reduction in HFrEF patients with DM for overweight and obese patients should be emphasized due to growing evidence showing that weight management may be achieved without increasing cardiovascular mortality. Newly introduced glucose-lowering agents such as sodium-glucose co-transporter 2 inhibitors and glucagon-like peptide-1 receptor agonists have also been shown to aid weight loss and improve cardiovascular outcomes.^[30–35]^ Sodium-glucose co-transporter 2 inhibitors have demonstrated their efficacy in patients with HFrEF in Dapagliflozin in Patients with Heart Failure and Reduced Ejection Fraction trial and Cardiovascular and Renal Outcomes with Empagliflozin in Heart Failure trial trials.^[36,37]^ Currently, the Taiwanese guideline recommends SGLT-2 inhibitors in obese HFrEF patients with or without DM as the first-line therapy.^[38]^

This study had several limitations. First, the patients included in this study were hospitalized patients, which means that this population may have advanced disease status compared to the general HF population. This analysis only enrolled patients in Taiwan; thus, the findings may not be directly applicable to all other Asian countries. Second, patient data on the metabolic control of diabetes were not collected, and glycemic control may affect HF outcomes. Third underweight patients were excluded from this study, which may have resulted in selection bias. Fourth, confounders including natriuretic peptide levels, troponin levels, body fat, and lean mass were not available in the registry data for analysis. Finally, the relatively short follow-up period and small sample size may have underestimated the association between survival and BMI.

## 5 Conclusion

In patients with HFrEF without DM, BMI was a significant predictor of survival. However, in patients with HFrEF with DM, BMI was not a significant predictor of survival. Both DM and higher BMI are associated with higher mean LA size, and HFrEF patients with higher BMI were less likely to have severe mitral regurgitation.

## Acknowledgment

The authors also express our gratitude and appreciation to the physicians and nurses participating in the TSOC-HFrEF registry.

The Principal investigators are: Kuei-Chuan Chan, Chung-Shan Medical University Hospital; Heng-Chia Chang, Taipei Tzu-Chi Hospital; Kuan-Cheng Chang, China Medical University Hospital; Zhih-Cherng Chen, Chi-Mei Hospital; Shyh-Ming Chen, Kaohsiung Chang-Gung Memorial Hospital; Pao-Hsien Chu, Linkou Chang-Gung Memorial Hospital; Chih-Hsin Hsu, National Cheng-Kung University Hospital; Jin-Long Huang, Taichung Veterans General Hospital; Jen yuan Kuo, Mackay Memorial Hospital; Jiunn-Lee Lin, National Taiwan University Hospital; Wei-Shiang Lin, Tri-Service General Hospital; Guang-Yuan Mar, Kaohsiung Veterans General Hospital; Kou-Gi Shyu, Shin-Kong Wu Ho-Su Memorial Hospital; Shih-Hsien Sung, Taipei Veterans General Hospital; Wei-Kung Tseng, E-Da Hospital; Wen-Chol Voon, Kaohsiung Medical University Chung-Ho Memorial Hospital; Ji-Hung Wang, Hualien Tzu-Chi General Hospital; Ming-Shien Wen, Linkou Chang-Gung Memorial Hospital; Chih-Cheng Wu, National Taiwan University Hospital, Hsinchu Branch; Yen-Wen Wu, Far Eastern Memorial Hospital; Ning-I Yang, Keelung Chang-Gung Memorial Hospital; Wei-Hsian Yin, Cheng-Hsin General Hospital.

## Author contributions

**Conceptualization:** Yu Ying Lu, Pao-Hsien Chu.

**Data curation:** Yu Ying Lu.

**Formal analysis:** Yu Ying Lu.

**Software:** Chieh-Yu Chang.

**Writing – original draft:** Yu Ying Lu.

**Writing – review & editing:** Victor Chien Chia Wu, Chien-Te Ho, Pao-Hsien Chu.
